# Tumor-infiltrating lymphocytes as predictive biomarkers in neoadjuvant treatment of HER2-positive breast cancer

**DOI:** 10.1093/oncolo/oyaf054

**Published:** 2025-04-24

**Authors:** Oğuzcan Kınıkoğlu, Yunus Emre Altıntaş, Anıl Yıldız, Goncagül Akdağ, Hamit Bal, Zeynep Yüksel Yaşar, Uğur Özkerim, Hacer Şahika Yıldız, Sıla Öksüz, Salih Tünbekici, Akif Doğan, Deniz Işık, Alper Yaşar, Tuğba Başoğlu, Heves Sürmeli, Hatice Odabaş, Nedim Turan

**Affiliations:** Kartal Dr. Lütfi Kirdar City Hospital, Health Science University, Department of Medical Oncology, Istanbul, Turkey; Kartal Dr. Lütfi Kirdar City Hospital, Health Science University, Department of Medical Oncology, Istanbul, Turkey; Istanbul University Oncology Institute, Department of Medical Oncology, Istanbul, Turkey; Kartal Dr. Lütfi Kirdar City Hospital, Health Science University, Department of Medical Oncology, Istanbul, Turkey; Kartal Dr. Lütfi Kirdar City Hospital, Health Science University, Department of Medical Oncology, Istanbul, Turkey; Kartal Dr. Lütfi Kirdar City Hospital, Health Science University, Department of Medical Oncology, Istanbul, Turkey; Kartal Dr. Lütfi Kirdar City Hospital, Health Science University, Department of Medical Oncology, Istanbul, Turkey; Kartal Dr. Lütfi Kirdar City Hospital, Health Science University, Department of Medical Oncology, Istanbul, Turkey; Kartal Dr. Lütfi Kirdar City Hospital, Health Science University, Department of Medical Oncology, Istanbul, Turkey; Ege University Faculty of Medicine, Department of Medical Oncology, Izmir, Turkey; Sancaktepe Şehit Prof. Dr. İlhan Varank City Hospital, Health Science University, Department of Medical Oncology, Istanbul, Turkey; Kartal Dr. Lütfi Kirdar City Hospital, Health Science University, Department of Medical Oncology, Istanbul, Turkey; Kartal Dr. Lütfi Kirdar City Hospital, Health Science University, Department of Medical Oncology, Istanbul, Turkey; Kartal Dr. Lütfi Kirdar City Hospital, Health Science University, Department of Medical Oncology, Istanbul, Turkey; Kartal Dr. Lütfi Kirdar City Hospital, Health Science University, Department of Medical Oncology, Istanbul, Turkey; Kartal Dr. Lütfi Kirdar City Hospital, Health Science University, Department of Medical Oncology, Istanbul, Turkey; Kartal Dr. Lütfi Kirdar City Hospital, Health Science University, Department of Medical Oncology, Istanbul, Turkey

**Keywords:** tumor infiltrating lymphocytes, HER2-positive breast cancer, neoadjuvant therapy, Ki67 suppression, pathological complete response

## Abstract

**Background:**

Tumor-infiltrating lymphocytes (TILs) have emerged as predictive biomarkers in HER2-positive breast cancer, correlating with treatment response and survival outcomes. This study evaluates the impact of TIL levels and Ki67 suppression on neoadjuvant therapy efficacy in this patient population.

**Materials and methods:**

A retrospective analysis of 136 HER2-positive breast cancer patients was conducted. Patients were stratified by TIL levels, and clinical outcomes, including Ki67 expression, pathological complete response (pCR), and disease-free survival (DFS), were assessed.

**Results:**

High TIL levels (≥ 40%) were significantly associated with higher pCR rates (60.32% vs. 39.73%, *P* = .02) and with TIL ≥ 10% greater Ki67 suppression. In patients with low TIL levels, high Ki67 expression correlated with better pCR rates (57.1% vs 30.8%, *P* = 0.010), while in high TIL patients, no significant difference was observed between high and low Ki67 groups (*P* = 0.317). A trend toward improved DFS was noted in the high TIL group, with 3-year survival rates of 91.9% vs. 80.7% in the low TIL group, though this was not statistically significant (*P* = .062).

**Conclusion:**

TIL levels are robust predictors of pCR and Ki67 suppression in HER2-positive breast cancer, particularly in patients with high initial TILs. These findings highlight the potential for integrating TIL evaluation into personalized treatment strategies to optimize neoadjuvant therapy outcomes. Further research is warranted to validate these results and explore underlying mechanisms.

**Implications for practice**

TIL assessments can enhance personalized treatment strategies in HER2-positive breast cancer by identifying patients likely to achieve better responses and long-term outcomes. High TIL levels correlate with higher pCR rates and greater Ki67 suppression, guiding more effective neoadjuvant therapies. Standardizing TIL evaluation in pathology reports can improve prognostic accuracy and treatment monitoring, ultimately leading to better patient outcomes. Integrating TIL analysis into routine clinical practice offers significant potential for optimizing treatment and improving survival rates in HER2-positive breast cancer patients.

## Introduction

Tumor-infiltrating lymphocytes (TILs) have gained significant attention in oncology due to their critical role in the immune response against cancer. These immune cells infiltrate tumor tissues, reflecting the host’s immune reaction to the malignancy.^1^ In the context of human epidermal growth factor receptor 2 (HER2)-positive breast cancer, TILs are emerging as important biomarkers for both prognosis and treatment response.^2^ Studies have demonstrated that high TIL levels are associated with better survival outcomes in patients with advanced HER2-positive breast cancer treated with therapies such as trastuzumab and pertuzumab. Specifically, increased TILs are linked to improved overall survival (OS), suggesting their significant role in enhancing the efficacy of HER2-targeted treatments.^3^

HER2-positive breast cancer is known for its aggressive nature and poorer prognosis compared to other breast cancer subtypes. However, advancements in targeted therapies, particularly those that inhibit the HER2 receptor, have dramatically improved patient outcomes. Neoadjuvant treatment has become a cornerstone in managing early-stage HER2-positive breast cancer.^4^ The role of TILs in neoadjuvant treatment is particularly noteworthy.^5^ Higher levels of TILs within the tumor microenvironment are associated with better responses to neoadjuvant therapies and improved patient outcomes.^6^ For instance, research from the NeoALTTO trial indicates that elevated TIL levels correlate with higher pathological complete response (pCR) rates and better event-free survival in patients receiving lapatinib and trastuzumab.^7^ This suggests that TILs can serve as predictive biomarkers, helping to optimize treatment plans for better efficacy.

Moreover, TILs have demonstrated their prognostic value in various studies. The FinHER trial, for example, showed that patients with high TIL levels benefited more from trastuzumab, underlining the interplay between immune response and HER2-targeted therapies.^8^ This insight opens avenues for more personalized treatment strategies, where the presence and density of TILs can guide therapeutic decisions, potentially leading to more favorable outcomes.

Additionally, TILs provide prognostic information in the context of residual disease after neoadjuvant therapy. Assessing TIL levels in residual tumors can offer valuable insights into the patient’s prognosis and inform subsequent treatment strategies.^9^ This emphasizes the importance of TILs as static biomarkers and dynamic indicators of treatment response and disease progression.

TILs are crucial in the neoadjuvant treatment landscape of HER2-positive breast cancer. Their presence is strongly associated with improved therapy responses and better prognoses; therefore, in this study, we aimed to investigate whether patients exhibiting elevated TIL levels demonstrate significantly improved clinical outcomes when subjected to HER2-targeted neoadjuvant treatment.

## Material and methods

### Study design and patient selection

This study is a retrospective single-center analysis conducted at our medical oncology clinic. The study aimed to investigate the role of TILs in predicting response rates to neoadjuvant therapy in HER2-positive breast cancer patients.

**Figure 1.**
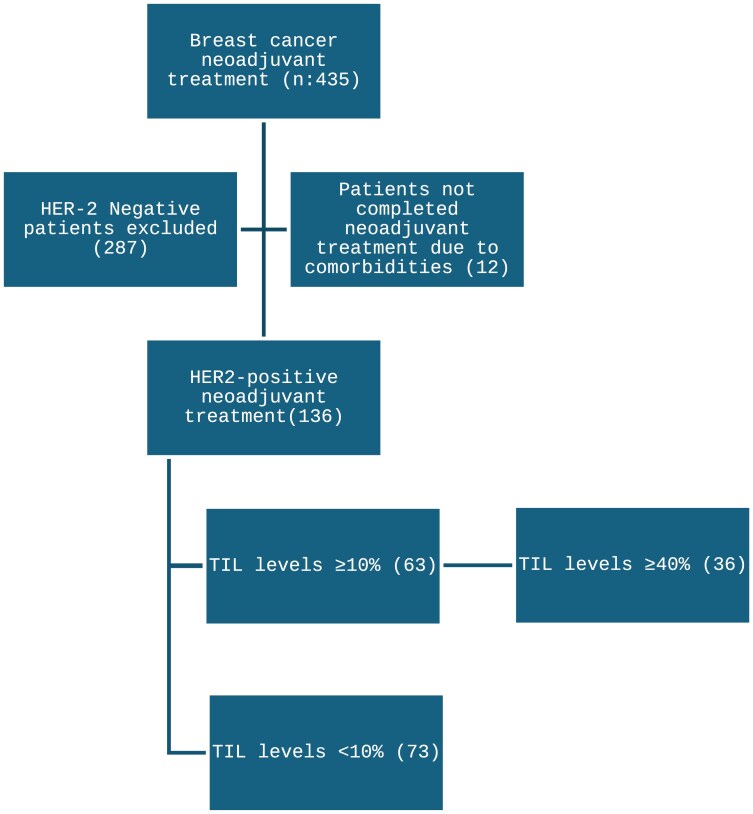
Patient selection and tumor infiltrating lymphocyte (TIL) distribution. The flow chart illustrates the selection process for TIL high and TIL low HER-2 positive breast cancer patients undergoing neoadjuvant treatment. Abbreviations: TIL, tumor infiltrating lymphocyte.

Initially, 435 patients who received neoadjuvant chemotherapy for breast cancer were reviewed. Among these, 153 patients were identified as HER2-positive, defined by either a HER2 score of 3+ by immunohistochemistry (IHC) or a HER2 score of 2+ confirmed by fluorescence in situ hybridization (FISH). Among these 153 patients, 136 had documented TIL scores in their pathology reports (Figure 1). Inclusion criteria were limited to those with early-stage HER2-positive breast cancer who received at least 4 cycles of anthracycline and anti-HER-2 treatment in a neoadjuvant setting and TIL evaluations. Exclusion criteria included patients with incomplete pathology reports or those who did not receive the specified neoadjuvant therapy regimen.

### Neoadjuvant therapy

All patients in the study received a neoadjuvant chemotherapy regimen consisting of anthracyclines and taxanes. In addition to anthracyclines, patients were treated with either taxane plus trastuzumab (T + T) or a combination of taxane plus trastuzumab (loading: 8 mg/kg/maintenance: 6 mg/kg) and pertuzumab (T + TP) (loading: 840 mg/maintenance: 420 mg) every three weeks intravenously based on the current clinical guidelines.

### TILs and tumor response evaluation

TIL levels were assessed using hematoxylin and eosin-stained tumor tissue sections. The evaluation followed the recommendations of the International TILs Working Group.^10^ Low (TILs: 0%-10%); moderate (TILs: 10%-40%); high (TILs: >40%). A single pathologist, blinded to patient outcomes, performed all TIL assessments. Previous reports utilized various cutoff points (10%, 30%, 50%, and 60%).^5,11-15^ However, there are no standardized cutoff points. Due to the limited number of patients, we compared patients with moderate and high TIL ratios to those with a low TIL ratio. Therefore, we took the value of 10% to ensure a more even distribution in our cohort.

To further investigate the impact of TIL thresholds on pCR and DFS, we created additional subgroups using a 40% cutoff. However, due to the limited number of patients with TIL levels >40% who also had residual tumor tissue available for evaluation, this analysis could not be performed for Ki67 levels. Consequently, the 10% cutoff was retained for Ki67 analyses.

The assessment of tumor response was carried out on specimens obtained during excision and divided into pCR and non-pCR. pCR was determined by the absence of invasive carcinoma and tumor thrombi in the lymphovascular channels within the breast, along with no evidence of metastasis in the axillary lymph nodes at the time of the surgical procedure (ypT0/ypTis and ypN0).

### Data collection and statistical analysis

Patient demographic data, treatment details, and clinical outcomes were extracted from medical records. The primary outcome was pCR, and the secondary outcome was DFS, defined as the time from the first initiation of neoadjuvant chemotherapy to recurrence or the last control date. Statistical analyses were performed using Python version 3.12.3 and SPSS version 27.0 (IBM Corp.). Descriptive statistics were used to summarize patient characteristics and treatment outcomes. Continuous variables were assessed for normality using the Shapiro–Wilk test. Group comparisons were performed using the Mann–Whitney *U* test for non-normally distributed data, while paired data were analyzed using the Wilcoxon signed-rank test. Categorical variables were analyzed using chi-square tests to evaluate associations between TIL levels and clinical outcomes. Multivariate analysis was conducted to adjust for potential confounding factors. Comparative analyses of initial TIL levels with postoperative pathological findings were also performed using appropriate non-parametric tests or independent *t*-tests, depending on the data distribution. A *P*-value of <0.05 was considered statistically significant.

### Ethics

This study was conducted in accordance with the ethical standards of our institution and the Helsinki Declaration. Ethical approval was obtained from the Institutional Review Board of Kartal Dr. Lütfi Kırdar City Hospital (date: 26.07.2024- decision no:2024/010.99/6/5). Informed consent was obtained from all patients before their inclusion in the study.

## Results

### Patient Characteristics and Demographics

This study included 136 patients diagnosed with HER2-positive breast cancer. The patients had an average age of 50.86 years (28-81 years). All patients were female, and 69 (50.74%) were postmenopausal. Eighty-two patients (60.29%) were HR-positive. Sixty-three patients (46%) had TIL levels ≥10%. Patient and disease characteristics are presented in Table 1.

**Table 1.** Patient characteristics and demographics.

Characteristic	Value, *n* (%)
Number of patients	136
Average age (range)	50.8 (28-81)
Menopausal status (postmenopausal)	69 (50.7)
Family history of breast cancer	18 (13.2)
ECOG performance status 0	119 (89.4)
HR-positive	82 (60.2)
HER2 3+	115 (84.6)
TILs ≥ 10%	63 (46.3)
TILs ≥ 40%	36 (26.5)

Abbreviations: ECOG PS, eastern cooperative oncology group performance score; HER2, human epidermal growth factor receptor; HR, hormone receptor; TIL, tumor-infiltrating lymphocyte; T + TP, taxane plus trastuzumab-pertuzumab..

In our analysis, we compared various clinical and pathological variables between patients with high and low levels of initial TILs. The median follow-up time was 33.6 months, from 0.43 to 104.8 months. The mean initial Ki67 level (48.4 vs 39.3) and higher tumor grade (G3, 65.1 vs 37.0) were significantly higher in the TIL ≥ 10 (both *P* < .01). No other significant differences were found in other variables between the TIL ≥ 10 and TIL < 10 groups (Supplementary Table S1). Similarly, using a TIL cutoff of 40 revealed no statistically significant differences across most parameters, except tumor grade, which was significantly higher in the TIL ≥ 40 group (*P* = 0.01) (Table 2).

**Table 2.** Comparison of variables between TIL ≥ 40 and TIL < 40 groups.

Variable	TIL ≥ 40 (37)	TIL < 40 (100)	*P*-value
Age (median)	49.5 (33-67)	51 (28-81)	.84
Postmenopausal (%)	17 (47.2)	52 (52)	.70
Family history (%)	5 (13.9)	13 (13)	.77
ECOG PS 0 (%)	33 (91.7)	86 (86)	.11
HR positive (%)	21 (58.3)	64 (64)	.93
HER2 3+ (%)	32 (88.9)	83 (83)	.59
Mean initial Ki67	48.1	42	NA
Median initial Ki67 (range)	45 (25-90)	40 (10-90)	.07
Tumor grade 3	25 (69.4)	43 (43)	.01
Tumor size (mean)	2.85	3.12	.88
Clinical node positivity (%)	32 (88.9)	84 (84)	.98
Neoadjuvant T + TP (%)	19 (52.8)	44 (44)	.43

Abbreviations: ECOG PS, eastern cooperative oncology group performance score; HR, hormone receptor; pCR, pathological complete response; T + TP, taxane plus trastuzumab-pertuzumab; TILs, tumor-infiltrating lymphocyte.

### Pathological complete response and predictive factors based on TIL levels

**Figure 2.**
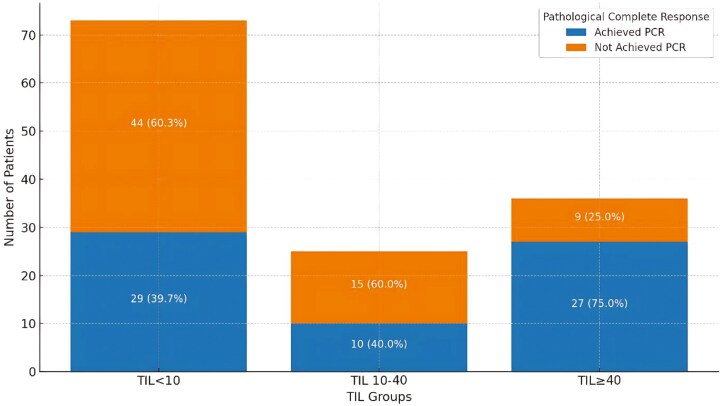
Pathological complete response by TIL groups. TIL Group (TIL < 10, TIL 10–40, TIL ≥ 40) vs pathological complete response. Patients with increasing TIL levels show progressively higher rates of complete pathological response, with the highest response observed in the TIL ≥ 40 group. Abbreviations: TIL, tumor-infiltrating lymphocytes; pCR, pathological complete response.

The median follow-up time was 33.6 months (0.43-104.8 months). A complete pathological response (pCR) was achieved in 67 patients (49.2%). Patients with high TIL (≥10) in their biopsy samples were more likely to achieve a pCR than those with low TIL levels (*P* = .026). Specifically, among patients with TIL ≥ 10, 38 patients (60.3%) achieved pCR, while 25 patients (39.7%) did not. In contrast, among patients with TIL < 10, 29 patients (39.7%) achieved pCR, while 44 patients (60.3%) did not. Additionally, a subgroup analysis using a higher cutoff of TIL ≥ 40 indicated an even stronger association with pCR: in the TIL ≥ 40 group, 27 patients (75%) achieved pCR, and only 9 patients (25%) did not, showing a significant difference compared to both the TIL 10-40 group (*P* = .008) and the TIL < 10 group (*P* = .0006). Among patients with TIL 10-40, 10 patients (40%) achieved pCR, while 15 (60%) did not. These findings emphasize that higher TIL levels, particularly above the threshold of 40, are strongly associated with achieving pCR (Figure 2).

A logistic regression analysis was conducted to further investigate the predictors of pCR, examining several pre-treatment variables, including CerbB2, ER, and PR status, clinical tumor size, clinical lymph node involvement, TIL ≥ 10, and TIL ≥ 40 levels. The analysis identified lymph node involvement (*P* = .004, OR = 1.805) and TIL ≥ 40 (*P* = .010, OR = 4.998) as significant predictors of achieving pCR. Specifically, patients with TIL ≥ 40 were nearly 5 times more likely to achieve pCR than those below this threshold. TIL ≥ 10, while indicative of an association with pCR in univariate analysis, did not reach statistical significance in the regression model (*P* = .470, OR = 1.453). These results underscore the potential of TIL ≥ 40 as a robust predictor of treatment response, suggesting that higher TIL levels might be particularly valuable for identifying patients likely to benefit from neoadjuvant therapy.

### Disease-free survival analysis by TIL cutoff values

For the TIL cutoff value of 10%, the mean DFS was 84.3 months (95% CI, 72.9-95.7) for patients with TIL < 10 and 89.0 months (95% CI, 77.3-100.8) for those with TIL ≥ 10. The overall mean DFS across both groups was 87.2 months (95% CI, 78.7-95.8). Notably, the median DFS was not reached in either group. The log-rank test comparing DFS between patients with TIL < 10 and TIL ≥ 10 did not demonstrate a statistically significant difference (*P* = .868), suggesting that TIL levels below or above 10 did not significantly impact DFS in this cohort.

**Figure 3.**
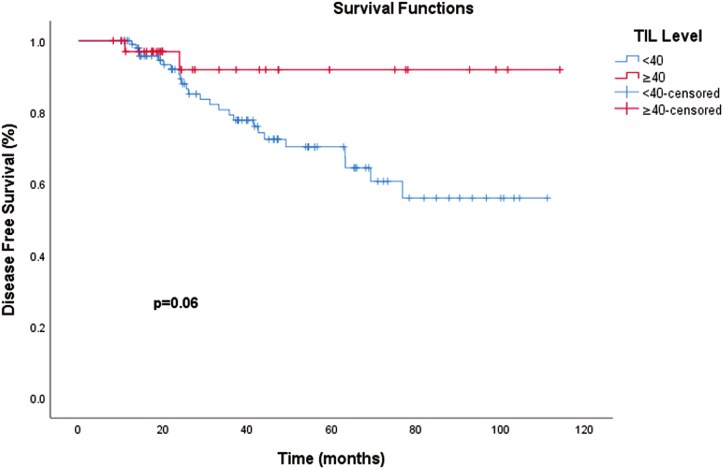
Disease-free survival (DFS) by TIL ≥ 40 vs TIL < 40. Disease-free survival (DFS) by TIL ≥ 40 vs TIL < 40. Patients with TIL ≥ 40 have a longer mean DFS (106.4 months) compared to those with TIL < 50 (80.9 months) and a higher 3-year survival rate (91.9% vs 80.7%), indicating a trend toward improved survival with higher TIL levels. Abbreviations: DFS, disease-free survival; TIL, tumor-infiltrating lymphocytes;.

In a subgroup analysis using a higher TIL cutoff of 40, the mean DFS was longer in the high TIL group. Patients with TIL ≥ 40 had a mean DFS of 106.4 months (95% CI, 95.9-116.9), while those with TIL < 40 had a mean DFS of 80.9 months (95% CI, 71.4-90.5), with median DFS again not reached. The log-rank test approached statistical significance (*χ*² = 3.483, *P* = .062), suggesting a potential trend toward improved DFS in patients with TIL ≥ 40, although this difference did not reach the conventional threshold for significance. Additionally, 3-year survival rates were higher for patients with TIL ≥ 40 (91.9%) compared to those with TIL < 40 (80.7%), suggesting a trend toward improved survival outcomes in the high TIL40 group (Figure 3).

### Impact of Ki67 levels and TIL presence on treatment response

The independent *t*-test comparing initial Ki67 levels between patients who achieved pCR and those who did not revealed a trend toward higher initial Ki67 levels in patients who achieved pCR. However, the difference was insignificant (mean Ki67, 46.7%; 95% CI, 42.1-51.3 vs mean Ki67, 40.8%; 95% CI, 36.2-45.3; *P* = .07) (Supplemental Figure S1).

**Figure 4.**
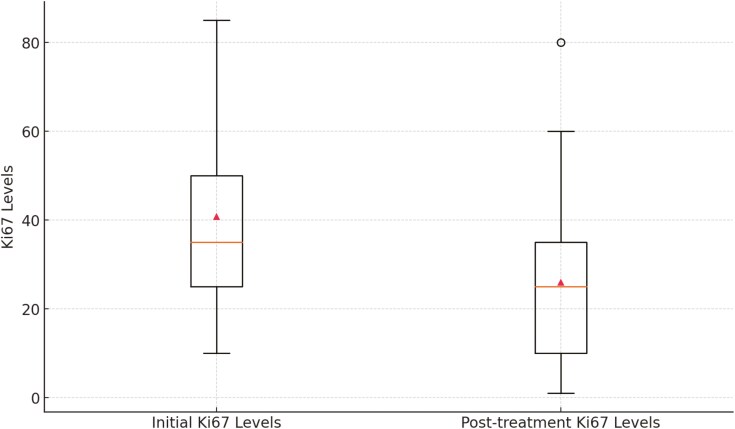
Ki67 suppression in whole cohort. Boxplot showing the significant reduction in Ki67 levels following treatment across the whole cohort, indicating effective Ki67 suppression.

When we further analyzed initial Ki67 levels and treatment outcomes, the paired *t*-test analysis revealed a highly statistically significant reduction in Ki67 levels from an initial mean of 40.80 to a final mean of 26.01 following treatment (95% CI, 10.4-19.1; *P* < .01), indicating effective Ki67 suppression in the overall patient cohort (Figure 4).

Further examination of the relationship between TIL levels and Ki67 suppression showed that, for patients with high TIL levels (≥10), there was a significant reduction in Ki67 levels after treatment (*P* < .01) (Supplementary Figure S2A). The paired *t*-test analysis for the low TIL level group also revealed a highly significant reduction in Ki67 levels following treatment (*P* < .01), indicating effective Ki67 suppression in patients with low TIL levels (Supplementary Figure S2B). Additionally, the comparison of Ki67 suppression between high TIL and low TIL groups revealed a statistically significant difference. The mean reduction in Ki67 levels was 22.52 (95% CI, 14.9-30.1) in the high TIL group and 10.39 (95% CI, 5.5-15.1) in the low TIL group. The mean percentage suppression of Ki67 levels was 44.93% in the high TIL group, compared to 27.91% in the low TIL group, further underscoring the greater effectiveness of treatment in the presence of TILs. This indicates that Ki67 suppression was significantly greater in patients with high TIL levels than those with low TIL levels (*P* < .01).

**Figure 5.**
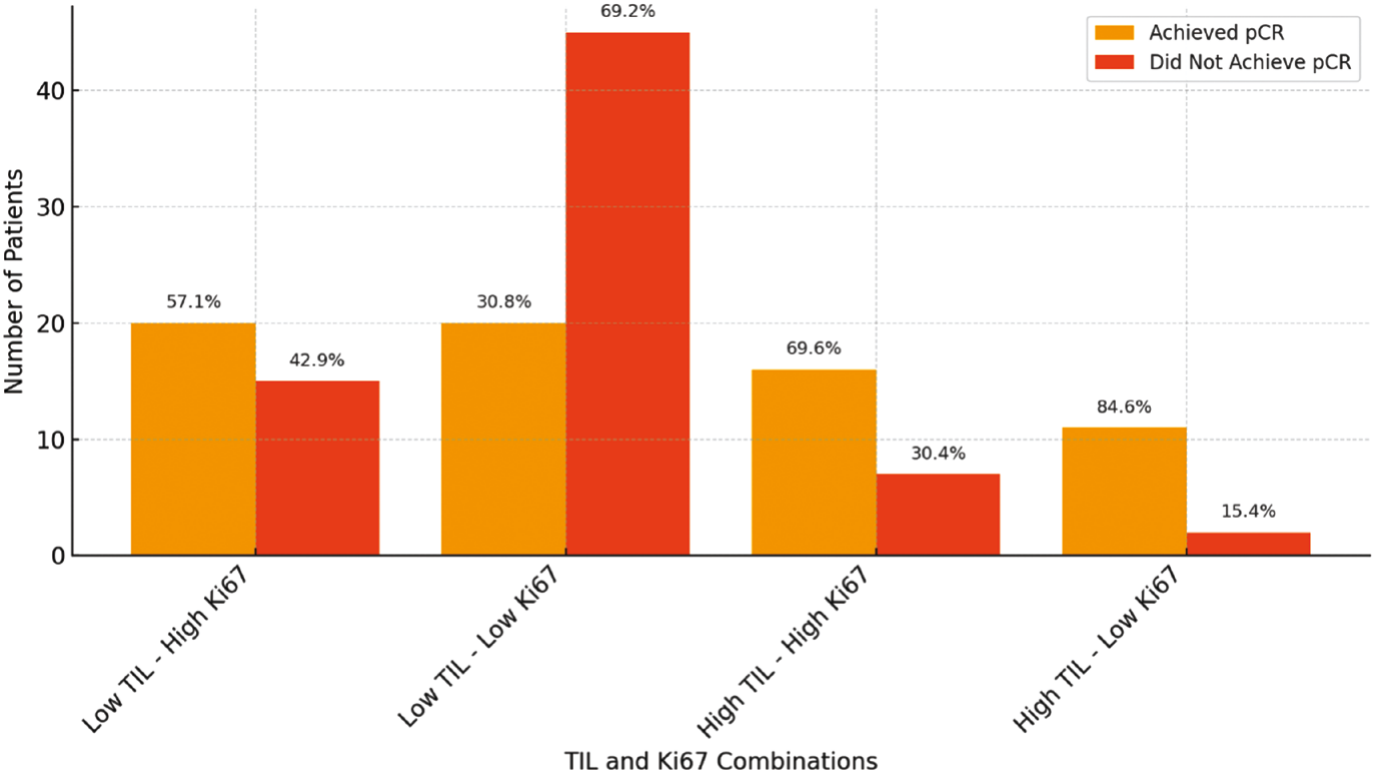
Pathological complete response (pCR) by TIL and Ki67 Levels. The bar plot shows pCR and non-pCR rates across TIL levels (low < 40%, high ≥ 40%) and Ki67 expression (high ≥ 40 and low < 40), with percentages annotated. Notable findings include higher pCR rates for high Ki67 in low TIL (57.1%) and high TIL groups (69.6%) and an 84.6% pCR rate for low Ki67 in high TIL.

For patients with low initial TIL levels (<40%), our analysis revealed a significant association between Ki67 expression and pCR. Among these patients, 20 with high Ki67 achieved pCR, while 15 did not. In contrast, 45 patients with low Ki67 did not achieve pCR, while 20 did. A chi-square test showed a statistically significant difference in pCR rates between high and low Ki67 groups within this subset (*P* = .010), indicating that high Ki67 expression is associated with an increased likelihood of achieving pCR in patients with low TIL levels. In contrast, for patients with high initial TIL levels(≥40%), the difference in pCR rates between high and low Ki67 groups was not statistically significant (*P* = .317). Among these patients, 16 with high Ki67 achieved pCR, while 7 did not. For patients with low Ki67, 11 achieved pCR, and 2 did not (Figure 5).

## Discussion

The findings from this study provide important insights into the role of TILs in predicting pCR in patients with HER2-positive breast cancer. Achieving a pCR was seen in 49.2% of patients in the whole population, consistent with results from other studies.^4,16-18^ For instance, Gianni et al reported a %45 pCR rate in the T + TP group,^4^ while Shao et al found a 39% pCR rate.^18^ A significant association was identified between TIL ≥ 10% in biopsy samples and the likelihood of achieving a pCR (*P* = .026). Notably, a subgroup analysis revealed that patients with TIL ≥ 40 demonstrated an even stronger association with pCR: 75% achieved pCR compared to only 25% who did not. This was significantly different from both the TIL 10–40 group (*P* = .008) and the TIL < 10 group (*P* = .0006). Furthermore, patients with TIL 10–40 showed intermediate results, with 40% achieving pCR while 60% did not. Logistic regression analysis further supported these findings, identifying TIL ≥ 40 as a robust predictor of pCR (*P* = .010, OR = 4.998), with patients nearly 5 times more likely to achieve pCR than those below this threshold. Although TIL ≥ 10 was associated with pCR in univariate analysis, it did not reach statistical significance in the regression model. These results highlight the potential of TIL ≥ 40 as a clinically significant biomarker for identifying patients most likely to benefit from neoadjuvant therapy, underscoring the importance of considering TIL levels in treatment planning. These findings reinforce the hypothesis that TILs play a critical role in mediating the tumor’s response to neoadjuvant therapy, as supported by other studies.^19-21^ For instance, in the NeoALTTO trial, it was observed that higher levels of TILs were associated with increased pCR rates across different treatment groups, including trastuzumab, lapatinib, or their combination. Specifically, TIL levels greater than 5% were linked to higher pCR rates, independent of the treatment group, with an adjusted odds ratio of 2.60.^7^ Cher-LOB trial also found a significant correlation between TIL levels and relapse-free survival (HR = 0.978 per 1% increase),^22^ alongside an association between pCR (OR 1.03, 95% CI, 1.02-1.05, *P* < .001 and OR 1.09, 95% CI, 1.04-1.15,*P* < .001, for Stromal TILs and Intratumoral-TILs, respectively).^23^ In the combined analysis of the CALGB 40601 and PAMELA trials, each 1% increase in TILs was associated with a higher likelihood of achieving pCR (OR = 1.01; 95% CI, 1.01-1.02; *P* = .02), and patients with TIL levels ≥ 40% had significantly better pCR rates compared to those below this threshold (OR = 2.29; 95% CI, 1.40-3.77; *P* = .02), emphasizing the critical prognostic role of TILs in HER2-positive breast cancer.^24^ Similarly, the analysis of the GeparQuattro and GeparQuinto trials demonstrated that lymphocyte-predominant breast cancer cases (≥ 60% TILs) had improved DFS when combined with pCR, defining a low-risk subgroup with significantly better outcomes (*P* = .039). Furthermore, in triple-positive breast cancer, TILs were shown to have greater prognostic significance for DFS (HR = 2.8; 95% CI, 0.987-7.909; *P* = .053), underscoring the differential impact of TILs based on hormone receptor status and their utility in refining risk stratification in HER2-positive breast cancer.^25^

These findings underscore the prognostic value of TILs in predicting treatment response in HER2-positive breast cancer. Further supporting this, a pooled analysis of 3771 patients treated with neoadjuvant therapy revealed that higher TIL concentrations were predictive of better responses across various breast cancer subtypes, including HER2-positive breast cancer. In this study, patients with high TIL levels (≥60%) had significantly higher pCR rates than those with low TIL levels. For instance, in the HER2-positive subtype, pCR was observed in 48% of patients with high TILs compared to 32% with low TILs (<10%).^11^ Similarly, the FinHER trial demonstrated that patients with high TIL levels had better responses to trastuzumab, with pCR rates of 60% in the high TIL (≥50%) group vs 36% in the low TIL group (*P* = .007).^8^ A meta-analysis of randomized controlled trials further corroborated these findings, demonstrating that high baseline TIL levels were associated with a significantly increased probability of achieving pCR in HER2-positive breast cancer patients treated with neoadjuvant chemotherapy plus trastuzumab and lapatinib, either alone or in combination. The odds ratio for pCR in patients with high TILs was 2.46, indicating a strong positive correlation between TIL levels and treatment response.^26^ Our findings are consistent with other existing literature, which highlights the prognostic value of TILs.^5-7,12,13,19,27^

High Ki67 levels often correlate with better pCR rates, making Ki67 a vital biomarker in evaluating treatment efficacy.^28^ An independent *t*-test indicated a trend toward higher initial Ki67 levels in the pCR group (*P* = .07). In another study, it was found that high TIL levels are associated with significantly higher relative Ki67 suppression from baseline compared to those with low TIL levels (64% vs 10%, *P* = .003) after neoadjuvant chemotherapy in hormone receptor-positive HER2-negative breast cancer.^29^ In our study, both high and low TIL groups (≥10% and <10%, respectively) experienced significant Ki67 reductions, with greater suppression in the high TIL group (44.9%; *P* < .01 for high TIL; 27.9%; *P* < .01 for low TIL).

Patients with high Ki67 expression and TIL levels have been shown to achieve significantly better DFS than those with low TIL levels.^30^ This suggests that the combination of high Ki67 expression and TIL presence generally enhances the efficacy of neoadjuvant chemotherapy, leading to improved outcomes in specific patient subgroups. In our study, among patients with low initial TIL levels (<40%), those with high Ki67 expression had significantly higher pCR rates compared to those with low Ki67 expression (57.1% vs 30.8%, *P* = .010). This indicates that, in the absence of robust immune infiltration, high proliferative activity reflected by Ki67 expression may act as a critical driver of response to chemotherapy.

However, in patients with high initial TIL levels (≥40%), no significant difference in pCR rates was observed between high and low Ki67 groups (*P* = .317). This finding may be influenced by the relatively small sample size in this subgroup, limiting statistical power. Additionally, the high baseline immune activity in these patients may overshadow the impact of Ki67, suggesting that immune-mediated mechanisms rather than proliferative activity dominate the response to treatment. Another potential explanation is that high TIL levels may already optimize chemotherapy response, leaving limited room for further improvement based on Ki67 expression alone. Further research with larger cohorts is warranted to confirm these findings and explore underlying biological mechanisms.

Liefaard et al. reported that in patients with TIL scores ≥ 60%, DFS was significantly higher than for patients with TIL scores < 60% (*P* = .041), with a 3-year DFS of 100% (95% CI, 94.3-100) and 92.3% (95% CI, 89.5-95.2), respectively.^31^ This finding underscores the potential of high TIL levels as a prognostic factor for better long-term outcomes. In our study, the analysis revealed varying impacts of TIL levels on DFS depending on the cutoff values used. For a TIL cutoff of 10%, the mean DFS was 84.3 months (95% CI, 72.9-95.7) for patients with TIL < 10 and 89.0 months (95% CI, 77.3-100.8) for those with TIL ≥ 10%, with no statistically significant difference observed between the groups (*P* = .868). This suggests that TIL levels below or above 10% may not significantly influence DFS in this cohort. However, subgroup analysis using a higher cutoff of 40% showed a more pronounced difference. Patients with TIL ≥ 40 had a mean DFS of 106.4 months (95% CI, 95.9-116.9), compared to 80.9 months (95% CI, 71.4-90.5) for those with TIL < 40. Although the log-rank test did not reach statistical significance (*P* = .062), the results suggest a trend toward improved DFS in the high TIL group, with 3-year survival rates of 91.9% for patients with TIL ≥ 40% compared to 80.7% for those with TIL < 40%.

These findings align with the suggestion that higher TIL levels may be associated with better early and long-term outcomes. The lack of statistical significance in our results, particularly with the 10% cutoff, may be attributed to the relatively short follow-up period and limited sample size. However, the observed trends, especially with the higher cutoff of 40%, highlight the potential prognostic utility of TIL levels in predicting survival outcomes. More extensive studies with larger cohorts and extended follow-up periods are needed to confirm these findings and establish the prognostic thresholds for TIL levels.

The association between TIL levels and Ki67 suppression emphasizes the importance of considering immune response indicators when evaluating tumor behavior and therapeutic outcomes. Patients with high TIL levels not only show better treatment response as evidenced by Ki67 reduction but also exhibit initially higher proliferative activity, which could indicate a more aggressive tumor phenotype responsive to immune-mediated interventions. These insights can guide personalized treatment strategies, optimizing therapeutic efficacy by leveraging the immune context of the tumor microenvironment. Overall, these findings underscore the prognostic significance of TILs in HER2-positive breast cancer, highlighting their potential utility in guiding therapeutic decisions and improving patient outcomes. Future studies should further elucidate the mechanisms underlying the relationship between TILs and treatment response, potentially paving the way for more tailored and effective treatment strategies.

Emerging evidence in triple-negative breast cancer highlights the importance of TILs as an essential metric for determining the risk-of-recurrence profile. A similar approach is increasingly relevant in HER2-positive breast cancer, where pCR is a key prognostic indicator associated with favorable outcomes.^32^ In HER2 + disease, TILs may serve as a valuable marker for identifying patients likely to achieve pCR and, consequently, better survival outcomes. This suggests that integrating immune metrics, specifically TIL assessments, could enhance the accuracy of recurrence risk stratification in HER2 + patients. By evaluating TILs alongside traditional clinical markers, clinicians could better personalize therapeutic strategies, optimizing outcomes by identifying those most likely to benefit from intensive neoadjuvant or immune-augmented approaches.

This study has several limitations that should be acknowledged to provide context for interpreting the results. Firstly, the retrospective design of the study introduces potential biases, particularly in data collection and interpretation. DFS was scored based on medical records and at the investigator’s discretion, which, despite efforts to ensure consistency through independent reviews, may still introduce variability in outcomes assessment.

Secondly, the study’s sample size, particularly in subgroup analyses, limits the statistical power to detect significant differences, especially for the higher TIL cutoff group (≥40%). This constraint necessitated the use of a lower TIL cutoff (≥10%) for certain analyses, such as Ki67 suppression, which may affect the generalizability of the findings.

Another limitation is the lack of standardized TIL cutoff thresholds across studies. While we introduced a 40% threshold to align with TIL study group, variability in TIL categorization across studies complicates direct comparisons. This highlights the need for standardization in TIL evaluation to ensure consistency and reproducibility in future research.

The relatively short follow-up period is another limitation, particularly for DFS analysis, which may not fully capture long-term outcomes. Longer follow-up is necessary to better assess the prognostic value of TIL levels and their relationship with DFS in HER2-positive breast cancer.

Despite these limitations, this study provides valuable insights into the role of TILs as predictive and prognostic biomarkers in HER2-positive breast cancer, contributing to the growing body of evidence supporting their clinical utility. Future prospective studies with larger cohorts, standardized methodologies, and extended follow-up periods are warranted to validate and expand upon these findings.

Future studies with larger prospective cohorts are warranted to validate these findings and further elucidate the mechanisms underlying the observed associations.

## Supplementary Material

oyaf054_suppl_Supplementary_Figures_1

oyaf054_suppl_Supplementary_Figures_2

oyaf054_suppl_Supplementary_Tables_1

## Data Availability

“The data presented in this study are available on request from the corresponding author.”
