# Supplementary material for: Tumor-infiltrating lymphocytes as predictive biomarkers in neoadjuvant treatment of HER2-positive breast cancer
Source: Oncologist. 2025 Apr 24;30(4):oyaf054. doi: 10.1093/oncolo/oyaf054 (PMC12019226; doi:10.1093/oncolo/oyaf054)
Supplement: oyaf054_suppl_Supplementary_Figures_1 [file oyaf054_suppl_supplementary_figures_1.docx]

**Supplemental Figure 1:** Comparison of Initial Ki67 Levels between pCR Achieved and Not Achieved Groups


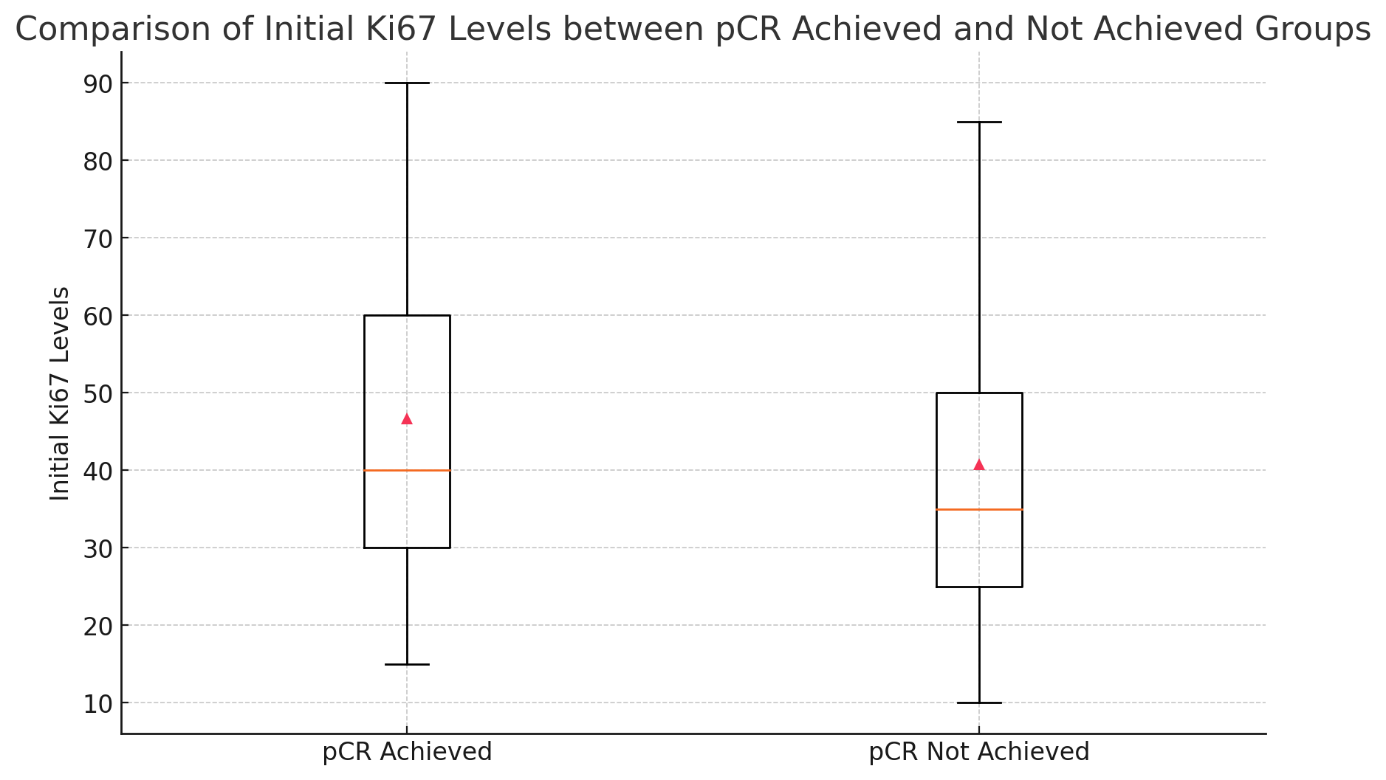


**Supplemental Figure 1.** Boxplot comparing initial Ki67 levels between patients who achieved pathological complete response and those who did not, showing a trend towards higher initial Ki67 levels in the pCR group. Abbreviation: pCR, pathological complete response
