# Supplementary material for: Tumor-infiltrating lymphocytes as predictive biomarkers in neoadjuvant treatment of HER2-positive breast cancer
Source: Oncologist. 2025 Apr 24;30(4):oyaf054. doi: 10.1093/oncolo/oyaf054 (PMC12019226; doi:10.1093/oncolo/oyaf054)
Supplement: oyaf054_suppl_Supplementary_Figures_2 [file oyaf054_suppl_supplementary_figures_2.docx]

**Supplemental Figure 2:** Ki67 Suppression in High TIL and Low TIL Groups

A B


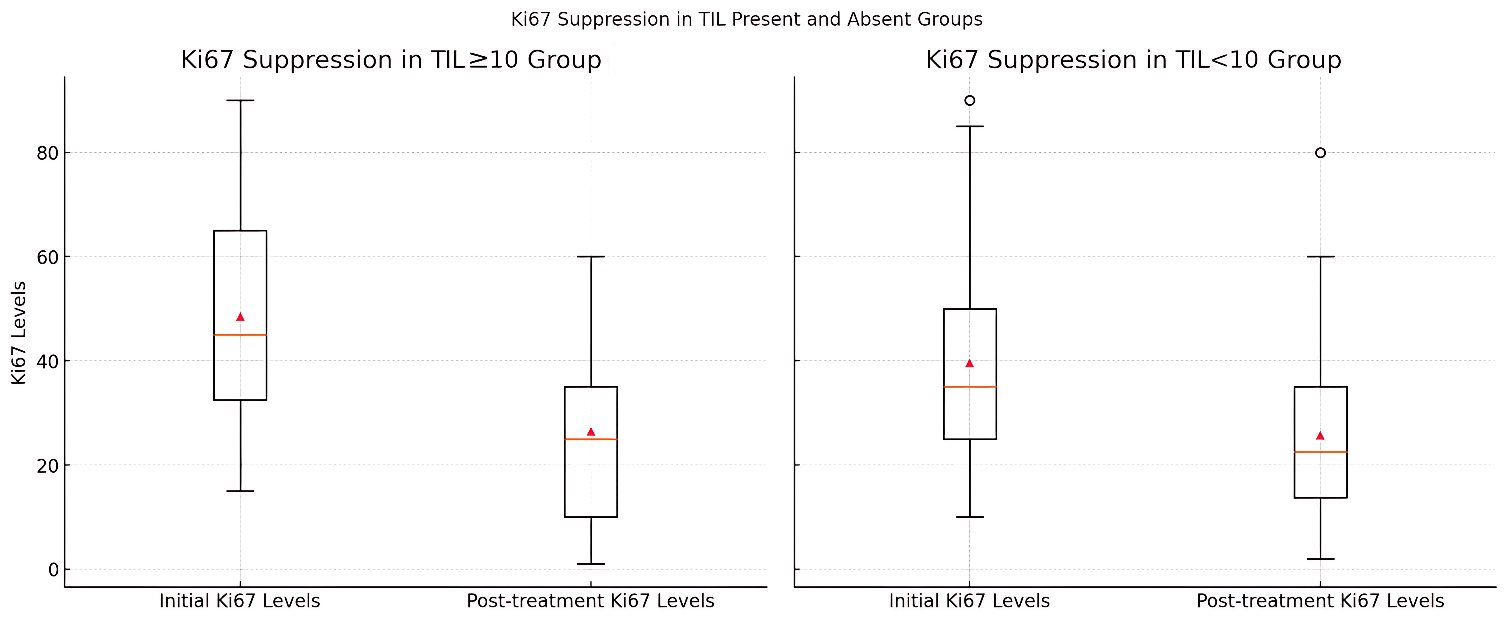


**Supplemental Figure 2.** Bar plot comparing the mean Ki67 suppression between high TIL(≥10) (A) and low TIL(<10) (B) groups, showing significantly greater suppression in the high TIL group—abbreviations: TIL, tumor-infiltrating lymphocyte.
