# Supplementary material for: Tumor-infiltrating lymphocytes as predictive biomarkers in neoadjuvant treatment of HER2-positive breast cancer
Source: Oncologist. 2025 Apr 24;30(4):oyaf054. doi: 10.1093/oncolo/oyaf054 (PMC12019226; doi:10.1093/oncolo/oyaf054)
Supplement: oyaf054_suppl_Supplementary_Tables_1 [file oyaf054_suppl_supplementary_tables_1.docx]

**Supplemental Table 1: Comparison of Variables Between TIL≥10 and TIL <10 Groups**

| **Variable** | **TIL≥10 (63)** | **TIL<10 (73)** | **P-Value** |
| --- | --- | --- | --- |
| Age (mean) | 50.14 | 51.50 | 0.437 |
| Postmenopausal (%) | 38 (60.3) | 31(42.4) | 0.739 |
| Family History (%) | 9 (14.2) | 9 (12.3) | 0.956 |
| ECOG PS 0 (%) | 62 (98.4) | 65(89) | 0.276 |
| HR Positive (%) | 44 (69.8) | 48 (65.7) | 0.151 |
| HER2 3+ (%) | 29 (46) | 43 (58.9) | 0.257 |
| Mean initial Ki67 (95% CI) | 48.4 (43.6%,53.4%) | 39.3 (35.4%, 43.7%) | NA |
| Median initial Ki67 (range) | 45 (15-90) | 35 (10-90) | <0.01 |
| Tumor Grade 3 | 41(65.1) | 27 (37) | <0.01 |
| Tumor Size (mean, cm) | 3.0 | 3.1 | 0.851 |
| Node Positivity (%) | 54 (85.7) | 62(84.9) | 0.98 |
| Neoadjuvant T+TP (%) | 31 (49.2) | 37 (50.6) | 0.86 |

**Supplemental Table 1.** Comparison of Variables Between High TIL and Low TIL Groups. Abbreviations: TILs, tumor-infiltrating lymphocyte; ECOG PS, eastern cooperative oncology group performance score; HR, hormone receptor; T+TP, taxane plus trastuzumab- pertuzumab; pCR, pathological complete response
